# Large-scale assessment of benthic communities across multiple marine protected areas using an autonomous underwater vehicle

**DOI:** 10.1371/journal.pone.0193711

**Published:** 2018-03-16

**Authors:** Renata Ferrari, Ezequiel M. Marzinelli, Camila Rezende Ayroza, Alan Jordan, Will F. Figueira, Maria Byrne, Hamish A. Malcolm, Stefan B. Williams, Peter D. Steinberg

**Affiliations:** 1 School of Life and Environmental Sciences, The University of Sydney, Sydney, New South Wales, Australia; 2 Sydney Institute of Marine Science and The University of Sydney, Sydney, New South Wales, Australia; 3 Australian Institute of Marine Sciences, Townsville, Australia; 4 Centre for Marine Bio-Innovation & Evolution and Ecology Research Centre, School of Biological, Earth and Environmental Sciences, University of New South Wales, Sydney, New South Wales, Australia; 5 Singapore Centre for Environmental Life Sciences Engineering, Nanyang Technological University, Singapore; 6 New South Wales Department of Primary Industries, Port Stephens, New South Wales, Australia; 7 School of Medical Sciences, The University of Sydney, Sydney, New South Wales, Australia; 8 New South Wales Department of Primary Industries, Coffs Harbour, New South Wales, Australia; Council for Scientific and Industrial Research, INDIA

## Abstract

Marine protected areas (MPAs) are designed to reduce threats to biodiversity and ecosystem functioning from anthropogenic activities. Assessment of MPAs effectiveness requires synchronous sampling of protected and non-protected areas at multiple spatial and temporal scales. We used an autonomous underwater vehicle to map benthic communities in replicate ‘no-take’ and ‘general-use’ (fishing allowed) zones within three MPAs along 7^o^ of latitude. We recorded 92 taxa and 38 morpho-groups across three large MPAs. We found that important habitat-forming biota (e.g. massive sponges) were more prevalent and abundant in no-take zones, while short ephemeral algae were more abundant in general-use zones, suggesting potential short-term effects of zoning (5–10 years). Yet, short-term effects of zoning were not detected at the community level (community structure or composition), while community structure varied significantly among MPAs. We conclude that by allowing rapid, simultaneous assessments at multiple spatial scales, autonomous underwater vehicles are useful to document changes in marine communities and identify adequate scales to manage them. This study advanced knowledge of marine benthic communities and their conservation in three ways. First, we quantified benthic biodiversity and abundance, generating the first baseline of these benthic communities against which the effectiveness of three large MPAs can be assessed. Second, we identified the taxonomic resolution necessary to assess both short and long-term effects of MPAs, concluding that coarse taxonomic resolution is sufficient given that analyses of community structure at different taxonomic levels were generally consistent. Yet, observed differences were taxa-specific and may have not been evident using our broader taxonomic classifications, a classification of mid to high taxonomic resolution may be necessary to determine zoning effects on key taxa. Third, we provide an example of statistical analyses and sampling design that once temporal sampling is incorporated will be useful to detect changes of marine benthic communities across multiple spatial and temporal scales.

## Introduction

Most Marine Protected Areas (MPAs) encompass a range of management zones that restrict specific activities, including ‘no-take zones’ (NTZ) that exclude fishing, through to zones that allow various extractive activities. Biological communities and environmental variables are often monitored through space (*e*.*g*. across zones) and time to evaluate effectiveness in achieving the marine area zoning objectives and inform adaptive management [1]. Small-scale experiments and observations (10s to 100s of meters) provide insights into important ecological processes and are useful to establish cause-effect relationships [2]. However, observations at experimental scales (either *ex-situ* or *in-situ* at scales of cm to a few meters) need to be coupled with large-scale (100s to 1000s of meters) studies to assess the effectiveness of MPAs across multiple spatial scales. These scales should be relevant to the expected biological responses [3].

MPAs are used to spatially reduce threats to species and ecological processes from anthropogenic activities, such as fishing or physical habitat disturbance [4–6]. These activities can have direct negative effects on entire biotic communities, especially through reductions in the abundance and size of targeted predatory species [5]. Habitat disturbance can lead to a reduction in ecosystem resistance [7] and resilience to climate change and other disturbances [8]. Biodiversity can also be altered indirectly due to changes in trophic interactions such as when removal of top predators has cascading effects on the benthos, resulting in changes of the entire assemblage [9]. Despite evidence that MPAs across the world, particularly NTZ, result in increased fish biomass and size structure [3, 10, 11], there is little information on their influence on benthic communities. Evaluating benthic species and community responses of individual MPAs is necessary to inform local adaptive management because (i) disturbances to benthic and habitat-forming species can reduce the amount of resources (food, shelter) available to other organisms in the system [5], and (ii) changes in higher trophic levels can have cascading effects on benthic communities [9].

The lack of synchronous, spatially explicit data at multiple scales across multiple MPAs is a major challenge in the evaluation of their effectiveness [12, 13], particularly in the short-term (<5–10 years), which is necessary for sound adaptive management. This is because representative sampling of biota underwater over large spatial scales can be resource intensive and often extremely difficult due to the time and depth constrains of diving [14]. This is particularly true for MPAs where use of destructive sampling tools (e.g. grabs, dredges) is precluded due to zoning regulations and so imagery is the preferred sampling method. An increasing amount of imagery is being collected using Automated Underwater Vehicles (AUVs) equipped with cameras, which can survey habitats below depths accessed by divers and can efficiently cover multiple large areas in a relatively short time-frame [15–17]. Importantly, AUVs can resurvey the same area accurately (<1 m precision) to detect temporal changes in benthic organisms [18] and so are particularly useful to assess short and long-term effects of stressors on benthic habitats, such as a shift in the dominant habitat-formers/morphological groups (i.e. from kelp dominated to sponge dominated) due to physical disturbance and climatic change [19, 20]. Constraints related to AUV technology should be considered; for example, operating AUVs is considerably more expensive and logistically challenging than other monitoring methods, such as diver-operated cameras [21]. However, the time/depth restrictions of diving (above) and the lack of spatial accuracy and depth consistency of other methods (e.g. towed video) make AUVs extremely useful in this context.

Another challenge in the evaluation of the effectiveness of MPAs is that detection of differences in average abundances of individual taxa and/or the entire community following protection typically requires long periods of time (> 20 years), especially for commercial fish species and thus on benthic biota they may influence [22]. However, human disturbances often affect not only average abundances of individual taxa, but also their variability, and such effects may occur in a shorter period of time [23, 24]. For instance, communities which are under lower stress levels, such as those in NTZ, can have less variation than communities exposed to more frequent disturbances from human activities [25]. By quantifying abundances of organisms in a community as well as how variable those communities are, monitoring programs may be able to detect short-term effects of protection from human disturbances in MPAs, thereby providing valuable information on the scales at which management actions would be most effective to maintain biodiversity.

In Australia, networks of MPAs have been implemented to represent and conserve the biological diversity of the continent’s underwater environments [26]. The Australian Integrated Marine Observing System (IMOS) supports an AUV facility with the objective of monitoring benthic assemblages at reference sites around the continent, encompassing tropical, subtropical and temperate reefs [27]. Here, we used IMOS AUV data to quantify benthic community structure, that is, the identity of taxa and their abundance in the community, as well as the variability in composition of the community, in NTZ and zones allowing most forms of fishing. AUV surveys were done across three spatial scales, from tens of meters to hundreds of kilometers, within three MPAs spanning 7° of latitude including a subtropical-temperate transition zone on the inner continental shelf (25–50 m depth) of Southeastern Australia. Benthic data were classified using the highest taxonomic resolution possible, as well as using morpho-taxa (*e*.*g*. encrusting coral), major taxa groups (Class) and morphological levels (*e*.*g*. massive forms). We used multivariate and univariate analyses to test whether similar patterns of community structure and composition were observed by using these broader classifications, and thus determine their suitability as surrogates for more detailed taxonomic data in the monitoring of MPAs [28]. The data provide the baseline needed to assess longer-term changes of epibenthic communities on rocky reefs along a subtropical to temperate transition zone, in MPAs with similar zoning arrangements. This is particularly important in this fast changing region, which is a global warming hotspot [29].

## Methods

### Study sites

**Fig 1:**
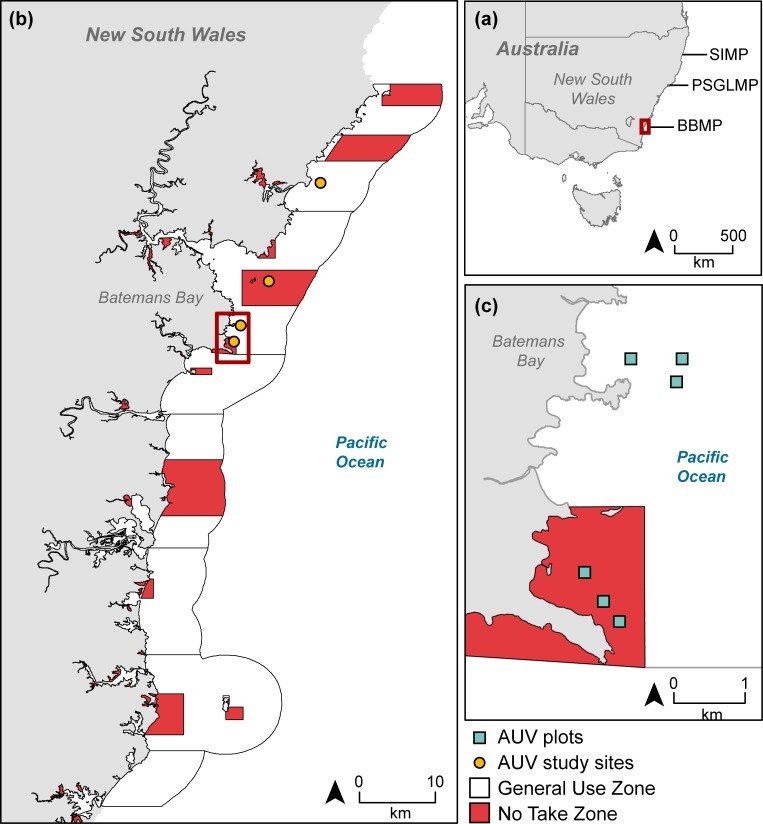
Study region in Southeastern Australia showing the three spatial extents considered in this study: (a) the three marine parks along the New South Wales coast: SIMP = Solitary Islands Marine Park, PSGLMP = Port Stephens Great Lakes Marine Park, and BMP = Batemans Bay Marine Park; (b) detail of one of the three marine parks, Batemans Bay Marine Park showing replicate study sites in no take zones and general use zones; (c) detail of two sites, one in a no take zone and one in a general use zone, showing three plots (25 x 25 m) where benthic community composition, structure and beta diversity were quantified. Not all sites and plot are shown in (a) and (b) due to the low spatial resolution but large spatial extent required to show the entire study area (7 degrees of latitude). In total there were 2X GUZ and 2X NTZ in PSGLMP and BMP, and 1x NTZ and 3x GUZ in SIMP. The zoning layers used in this Figure were made available by the NSW Department of Primary Industries under a CC BY licence.

The IMOS AUV facility and New South Wales (NSW) Department of Primary Industries initiated a long-term benthic monitoring program of three MPAs along the southeastern Australian coast: Solitary Islands Marine Park, Port Stephens-Great Lakes Marine Park and Batemans Bay Marine Park (Fig 1; see below). These MPAs have a similar design, with spatially interspersed replicate NTZ (named “sanctuary zones”) and General-Use Zones (GUZ) that allow all sorts of recreational fishing and most forms of commercial fishing, except for destructive fishing (dredging, longlining) although trawling is allowed in two of the three MPAs studied. This spatial mosaic allows an assessment of the benthic assemblages between different management areas across multiple spatial scales. The dominant habitat-forming organisms in these MPAs are large, canopy-forming brown macroalgae (*e*.*g*. the kelp *Ecklonia radiata)* and massive sponges (e.g. orange sand sponge or ball sponges), although scleractinian corals are dominant in shallow waters of the Solitary Islands, the northern-most MPA. In 2012, benthic assemblage structure (relative abundance and identity of taxa) and composition (identity only, presence/absence) were quantified on deep-water (25–50 m) rocky reefs at the three MPAs investigated here (Fig 1), which are briefly described below.

#### Solitary Islands Marine Park

The 720 km^2^ Solitary Islands Marine Park (29°55'18.11"S, 153°23'11.52"E) was initially established in 1991 with few small NTZ (<1% of the area) and was rezoned in 2002, expanding NTZ to ~12% of the total area. Solitary Islands is the northernmost MPA in this study and is located in a tropical-temperate transition zone. Here, benthic communities are highly diverse [30] and change with depth [31], with scleractinian coral-dominated communities above 25 m depth and sponge-dominated communities below 25 m. Macroalgal-dominated communities are also present close (within 1.5 km) to the mainland coast [29], but have have shown strong declines in the last ~15 years due to grazing by range-expanding tropical herbivorous fish and increased temperature [28].

#### Port Stephens-Great Lakes Marine Park

The 980 km^2^ Port Stephens-Great Lakes Marine Park (32°43'49.62"S, 152°13'30.14"E) was declared in 2005 and its zoning plan came into force in 2007, with NTZ representing ~18% of the total area. Port Stephens is located approximately 300 km south of the Solitary Islands and its rocky reefs are predominantly sponge dominated below 30 m depth, and macroalgal dominated in shallower depths [17]. Scleractinian corals are rare at Port Stephens.

#### Batemans Bay Marine Park

The 850 km^2^ Batemans Bay Marine Park (36°13' 11.67"S, 150°14' 48.79"E) was declared in 2006 and its zoning plan came into force in 2007 with NTZ representing ~19% of its area. Batemans Bay is the southernmost of the surveyed MPAs, located approximately 350 km south of Port Stephens, and is representative of a temperate reef. Most shallow (< 25 m) reefs are dominated by the kelp *E*. *radiata* or sea urchin barrens, and deeper reefs (> 25 m) generally dominated by sponges and other sessile invertebrates, although kelp can be found up to 40 m [17, 32, 33, 34].

### Sampling design

Within each MPA, replicate NTZ and GUZ sites were randomly selected using a randomly stratified design, resulting in similar (*e*.*g*. similar aspect, zoning, depth) available sites under each zoning type in each park. The number of sites varied among MPAs, with two NTZ and two GUZ sites in Port Stephens and Batemans Bay, and one NTZ and three GUZ in Solitary Islands. These were the maximum number of sites for each zoning type and MPA that could be surveyed based on logistics (*e*.*g*. travel time and distance, weather) and resources (*e*.*g*. shipping time) available at the time of sampling. The spatial design of zoning in MPAs in NSW enabled surveying sites from each zoning type that were spatially interspersed. Three (Port Stephens and Batemans Bay) or six (Solitary Islands) 625 m^2^ plots (25 x 25 m) of rocky reef were surveyed per site, with the exception of one GUZ site at Solitary Islands where seven plots were surveyed. Sites were at least 1 km apart within each park, while plots within sites were at least 200 m apart (Fig 1).

Surveys were conducted on rocky reefs previously mapped using swath acoustics [33, 34], in depths of 25–50 m (each site had plots across this depth range but a single plot only contained a range of approximately 5–10 m within the 25–50 m depth range, e.g. 25–35 m or 40–50 m). Using the swath acoustic maps, sites were identified to be representative of the area, with similar reef characteristics (i.e. slope and structural complexity), and dominated by macroalgae, principally *E*. *radiata*, turf algae or sessile invertebrates [30, 35]. All sites were surveyed within 3 months during late winter/spring of 2012 to reduce the influence of seasonal effects (Solitary Islands: 22–28 August, Port Stephens: 8–14 November, Batemans Bay: 27–29 November). These assemblages were surveyed using the IMOS AUV, which navigated at ~2 m above the seafloor taking high-resolution (1.5 MP), geo-referenced stereo pairs of images of the seafloor approximately every half second (2 pairs per second).

**Fig 2:**
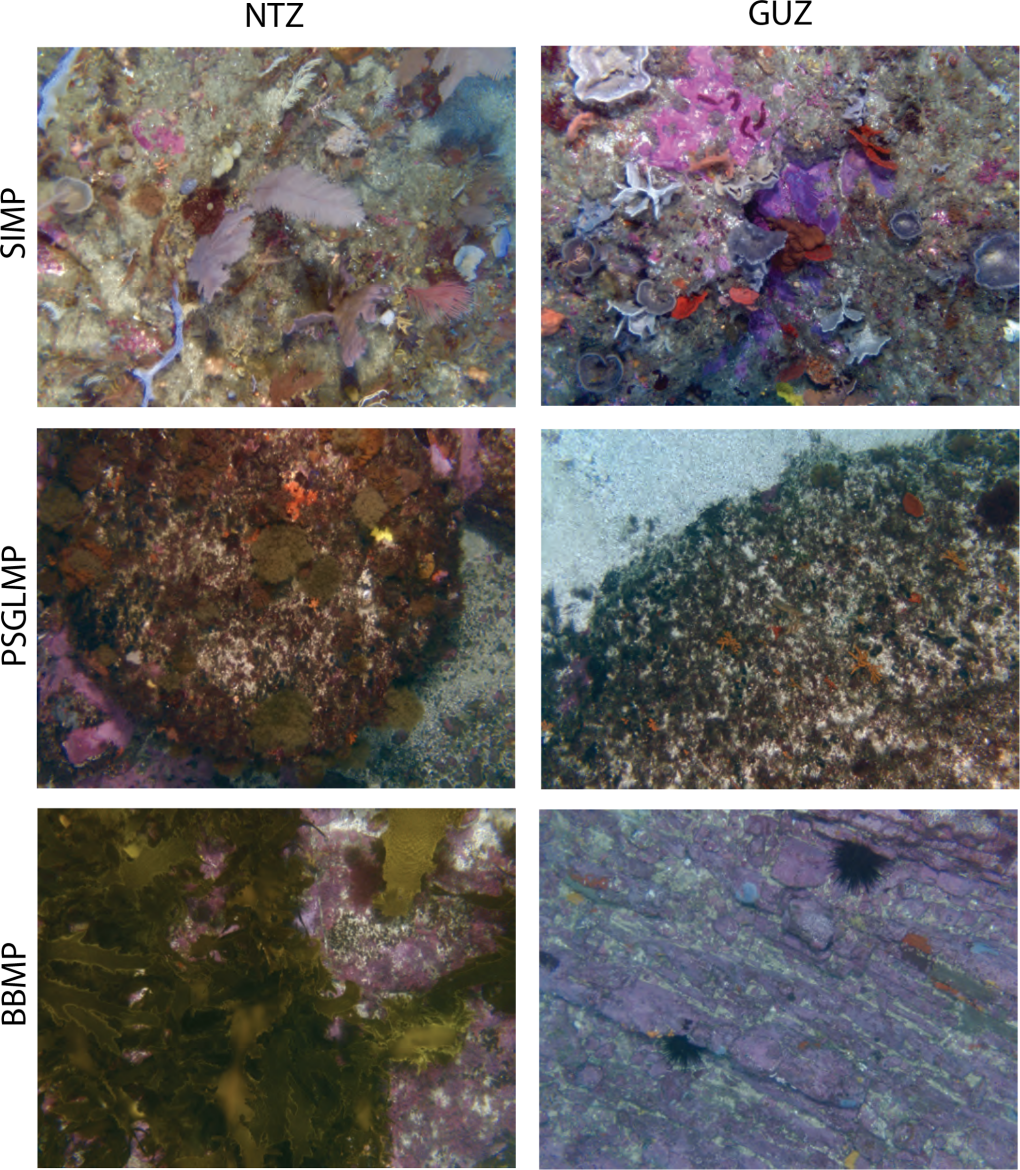
Example images from each MPA (SIMP = Solitary Islands Marine Park, PSGLMP = Port Stephens Greatl Lakes Marine Park, BBMP = Batemans Bay Marine Park) and each management zone (NTZ = No Take Zone, GUZ = General Use Zone).

Full coverage of each 625 m^2^ plot typically resulted in ~15,000 images, from which 50 spatially balanced images, each covering an area of ~2 m^2^, were selected using a generalized random tessellated stratified design with the R package ‘spsurvey’ [36, 37]. The number of images per plot (n = 50) was chosen based on a previous study by Bridge et al. [19], which used power analysis of changes in variance with increasing replication, from 5 to 50 images for a given plot, and which indicated negligible further reduction in residual standard error of percent cover for benthic categories (i.e. canopy forming macroalgae) above 30 AUV images per 625 m^2^ plot. Results of the power analysis also indicated little improvement in detectable effect sizes (given bootstrapped variance estimates, such as standard deviation, standard error, confidence interval and residual standard error) for image replication levels above 30 [19]. Thirty images (~2 m^2^ each) cover approximately 10% of the area of of each 625 m^2^ plot, while fifty images covered ~15%. Given the rarity of this data and to allow for a margin of error, we chose to include up to 50 images where the data was available. Some images were too dark to use due to shading by nearby reef structures, so the final number of images scored per plot ranged between 29 and 50, but at least 45 images were used in over 80% of the plots (Fig 2).

Twenty-five random points were overlaid on each image and taxa under each point were identified to the highest taxonomic resolution possible using Coral Point Count software with Excel extensions (CPCe) [38]. The national standard classification scheme, the Collaborative and Automated Tools for Analysis of Marine Imagery (CATAMI) Version 1.2 [39], was used to identify organisms to a taxonomic, morpho-taxa (*e*.*g*. encrusting coral), major group (Class) and morphological level (Table A in SI File) [39, 40], as higher-taxon data has been suggested as a suitable surrogate for species data in the assessment of MPA effectiveness [28].

### Data analyses

#### Multivariate analyses

Benthic assemblage multivariate data were analyzed using permutational multivariate analyses of variance [41] with the PERMANOVA add-on in PRIMER v6 [42]. For comparisons of benthic assemblages between zoning types and MPAs, ‘Zoning’ was a fixed factor with 2 levels (NTZ, GUZ), ‘MPAs’ was also fixed, with 3 levels (Solitary Islands, Port Stephens, Batemans Bay), and ‘Site’ was a random factor, nested in Zoning x MPAs, with 1 (NTZ at Solitary Islands), 2 (NTZ and GUZ at Batemans Bay and Port Stephens) or 3 (GUZ at Solitary Islands) levels. The replicates were the plots within each site (*n* = 3 for all Port Stephens and Batemans Bay sites; *n* = 6–7 for all Solitary Islands sites). Relative abundance of benthic classes per plot consisted of the averaged percent cover quantified over all images in a plot. Dissimilarity matrices based on the Bray-Curtis measure of square-root transformed relative abundances (providing information of the identity and abundance of the taxa present; ‘assemblage structure’) or on the Jaccard measure (presence/absence, providing information of the identity of the taxa present; ‘assemblage composition’) were generated for the analyses, which used 9,999 permutations of residuals under a reduced model. Analyses were done on individual taxa (identified to different taxonomic levels), morpho-taxa groups or morphological groups (e.g. massive), and major groups (Class).

Each analysis was run twice, either including or excluding abiotic substratum types (bare rock, cobbles, pebbles and unconsolidated substratum) to consider either total benthic cover or just biotic cover, focusing on biodiversity alone. Multivariate dispersion quantified as distances of replicate samples from group centroids was also compared between zones (pooled across all three MPAs) and MPAs (pooled across both zoning types) using PERMDISP in PRIMER v6 to test for differences in overall multivariate heterogeneity/dispersion of the communities [42, 43].

In addition, we compared compositional variability among zoning types and MPAs using Jaccard dissimilarities, which focus on variation in the identity of taxa/groups present in the community, calculated for each site (using all scored images in the plots in each site) in the R package ‘MBI’ [44]. Because our sampling design was unbalanced and different numbers of images could be scored per plot, the area within zoning types in some parks were sampled to a greater extent than others, particularly at Solitary Islands (c. 1802 m^2^ GUZ vs c. 592 m^2^ NTZ). The differences in overall area sampled can lead to biased estimates of compositional variation, so we modeled Jaccard dissimilarities as a function of area sampled (fitted first in the model to account for differences in sampling intensity), and zoning type, park and their interaction using a linear model in R. Test-statistics and p-values for each term in the model were obtained using the ‘anova’ function with Type I sums of squares. Non-metric multi-dimensional scaling (nMDS) was used as an ordination method to visualize multivariate patterns in benthic assemblages [45].

#### Univariate analyses

Univariate analyses on the effect of zone and MPAs on the abundance/prevalence of individual benthic taxa were also completed for the most prevalent (> 10% occurrence across all images; 16 taxa) and abundant groups (> 5% cover; 8 taxa, which overlapped with those found to be most prevalent). We used Generalized Linear Mixed Models (GLMM) assuming a binomial distribution [46] to test the effect of zoning type, MPAs and their interaction on the coverage and prevalence (data transformed to presence/absence per image) of these groups. Sites (within zones and MPAs) and plots (within sites) were fitted as random effects, and the replicates were the images (*n* = 29–50). Analyses were done using the ‘lme4’ package in R [47]. Assumptions of homogeneity and spatial independence were checked using residual plots in all final models [48]. Where effects on terms in the model with more than two levels were found, multiple comparison analyses were done using Tukey tests in the ‘multcomp’ package in R, adjusting for multiple testing [49]. Some groups were absent (or present in very low abundances, < 1% mean cover) in some MPAs; in these cases, data from those MPAs were excluded from analyses of those groups (Tables C and D in S1 File).

We also used a similarity of percentages analyses (SIMPER) [45] to determine the contribution of these most prevalent and abundant groups (above) and other taxa to the overall community dissimilarity between zones in each MPA (Table B in S1 File).

## Results

### Differences in community structure and composition

**Fig 3:**
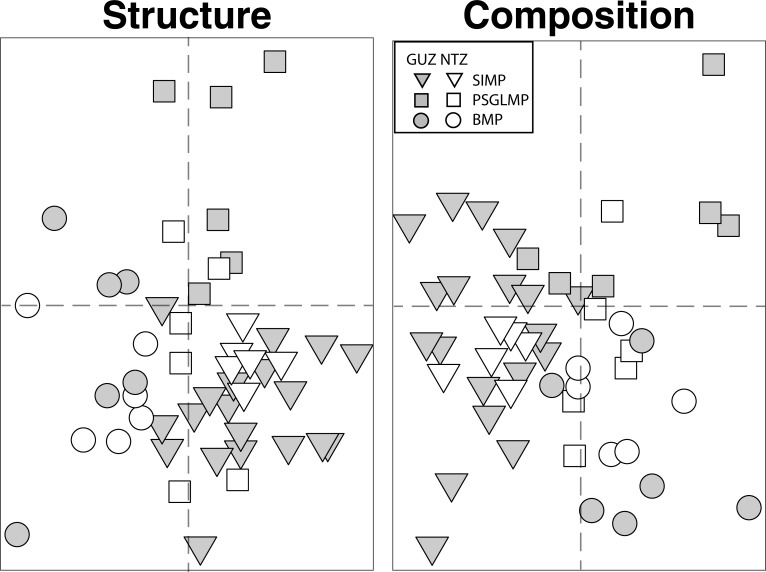
nMDS ordination based on (a) Bray-Curtis similarity measure for square-root transformed % cover (assemblage structure) or (b) Jaccard measure (presence/absence; assemblage composition) of benthic assemblages (all taxa) in no-take (NTZ; white symbols) and general use zones (GUZ; grey symbols), at 3 MPAs in NSW: Solitary Islands (SIMP; triangles), Port Stephens (PSGLMP; squares) and Batemans Bay MP (BMP; circles).

Overall, we found 104 taxa and 41 morpho-taxa (e.g. massive coral), belonging to 16 major groups (Class), as well as 14 morphological types (i.e. massive) in all the MPAs (Table A in S1 File). There were no differences in benthic community structure between NTZ and General Use Zones (GUZ) when considering all taxa (Fig 3, Table 1). There were, however, significant differences in community structure (identity and abundance of taxa/groups) among the three MPAs, despite significant variability among sites within MPAs (Fig 3, Table 1). A similar pattern was observed for major groups, morpho-taxa and morphological types, although in some cases community structure in Solitary Islands (30^o^ S) and Port Stephens (32^o^ S) did not differ from each other, but were significantly different from those in Batemans Bay (36^o^ S) (Table 1). The same overall pattern was observed when substratum types were excluded from analyses except for major groups, where no differences among MPAs were detected (Table 1). No differences in multivariate dispersion in community structure were found between zones or among MPAs (Table 2).

**Table 1:** PERMANOVA summary table for effect of zone on benthic community structure (a) and biodiversity (b) across three MPAs.

			+ Substrata						- Substrata					
			(a) Square-root %cover			(b) Presence/absence			(a) Square-root %cover			(b) Presence/absence		
	Source	*df*	MS	pseudo-*F*	*p*(perm)	MS	pseudo-*F*	*p*(perm)	MS	pseudo-*F*	*p*(perm)	MS	pseudo-*F*	*p*(perm)
**Taxa**	Zone	1	1807	1.02	0.45	1964	0.73	0.70	1658	0.88	0.56	1959	0.70	0.74
	MPAs	2	6232	3.45	**<0.01**	6701	2.46	**<0.01**	6640	3.45	**<0.01**	7229	2.54	**0.01**
	Z x M	2	1724	0.95	0.53	1875	0.69	0.85	1342	0.70	0.82	1935	0.68	0.88
	Si(ZxM)	6	1887	3.83	**<0.01**	2816	2.36	**<0.01**	2009	4.27	**<0.01**	2935	2.29	**<0.01**
	Residual	37	493			1192			471			1280		
			**SIMP≠PSGLMP≠BMP**			**SIMP = PSGLMP≠BMP**			**SIMP = PSGLMP≠BMP**			**SIMP≠PSGLMP≠BMP**		
**Major**	Zone	1	413	0.87	0.51	1344	1.31	0.28	259	0.61	0.71	1563	1.29	0.29
**Groups**	MPAs	2	1397	2.88	**0.03**	2057	1.97	0.10	925	2.15	0.06	2335	1.91	0.11
	Z x M	2	647	1.34	0.26	888	0.85	0.60	369	0.86	0.61	1061	0.87	0.59
	Si(ZxM)	6	502	2.66	**<0.01**	1073	1.98	**<0.01**	448	3.39	**<0.01**	1260	1.94	**<0.01**
	Residual	37	189			542			132			650		
			**SIMP = PSGLMP, PSGLMP≠BMP, SIMP = BMP**											
**Morpho-**	Zone	1	1372	1.12	0.37	1060	0.74	0.64	1389	0.99	0.46	1154	0.74	0.64
**groups**	MPAs	2	5007	4.01	**<0.01**	4664	3.19	**<0.01**	5500	3.85	**0.01**	4973	3.15	**0.01**
	Z x M	2	1253	1.00	0.47	1229	0.84	0.64	1058	0.74	0.74	1336	0.85	0.65
	Si(ZxM)	6	1303	3.88	**<0.01**	1512	2.32	**<0.01**	1495	4.66	**<0.01**	1632	2.30	**<0.01**
	Residual	37	336			652			321			710		
			**SIMP = PSGLMP≠BMP**			**SIMP≠PSGLMP = BMP**			**SIMP = PSGLMP≠BMP**			**SIMP≠PSGLMP = BMP**		
**Morph**	Zone	1	1196	1.65	0.18	487	1.03	0.43	1142	1.42	0.25	588	1.04	0.43
	MPAs	2	3652	4.94	**<0.01**	2849	5.94	**0.01**	3972	4.84	**0.01**	3403	5.90	**<0.01**
	Z x M	2	867	1.17	0.36	391	0.82	0.61	629	0.77	0.68	468	0.81	0.62
	Si(ZxM)	6	770	3.66	**<0.01**	496	2.39	**<0.01**	860	5.03	**<0.01**	597	2.37	**<0.01**
	Residual	37	211			207			171			251		
			**SIMP = PSGLMP≠BMP**			**SIMP = PSGLMP≠BMP**			**SIMP = PSGLMP≠BMP**			**SIMP≠PSGLMP≠BMP**		

(a) Bray-Curtis similarity measure for square-root transformed percent cover or (b) Jaccard measure (presence/absence) of benthic assemblages in no-take zones (NTZ) *vs* general use zones (GUZ) at 3 MPAs in New South Wales, Australia: Solitary Islands Marine Park (SIMP), Port Stephens-Great Lakes Marine Park (PSGLMP) and Batemans Bay Marine Park (BMP). Analyses were completed on all taxa, major groups, morpho-groups or morphology (morph), including (+) or excluding (-) cover of substrata (see Table A in S1 File). Zone (Z) was fixed (2 levels: NTZ *vs* GUZ), MPAs (M) was fixed (3 levels: SIMP, PSGLMP, BMP), Site was random, nested in ZxM. The replicates were the 2 5x 25 m plots in each site, which consisted of % covers averaged over 50 images per plot. Analyses used 9999 permutations of residuals under a reduced model.

**Table 2:** PERMDISP analyses of multivariate dispersion from group centroids of community structure and biodiversity.

		+ Substrata						- Substrata			
		(a) sqrt(%cover)			(b) P/A			(a) sqrt(%cover)		(b) P/A	
	Factor	*df*	pseudo-*F*	*p*(perm)		pseudo-*F*	*p*(perm)	pseudo-*F*	*p*(perm)	pseudo-*F*	*p*(perm)
**Taxa**	Zone	1, 47	4.52	0.06		7.03	**0.02**	3.81	0.08	4.92	0.05
	MPAs	2, 46	2.28	0.16		0.77	0.55	1.05	0.42	0.51	0.68
						**GUZ > NTZ**					
**Major**	Zone	1, 47	1.68	0.23		0.88	0.44	3.48	0.08	0.88	0.43
**Groups**	MPAs	2, 46	1.09	0.40		7.74	**<0.01**	0.34	0.75	8.18	**<0.01**
				**SIMP < PSGLMP = BMP**					**SIMP < PSGLMP = BMP**		
**Morpho-**	Zone	1, 47	3.92	0.08		2.94	0.20	3.76	0.10	2.06	0.21
**groups**	MPAs	2, 46	1.65	0.25		2.51	0.15	1.46	0.30	2.75	0.13
**Morphology**	Zone	1, 47	1.50	0.27		1.95	0.23	1.32	0.31	1.91	0.23
	MPAs	2, 46	2.38	0.13		6.67	**<0.01**	2.48	0.14	6.50	**<0.01**
				**SIMP > PSGLMP = BMP**					**SIMP > PSGLMP = BMP**		

(a) Bray-Curtis similarity measure for square-root transformed % cover or (b) Jaccard measure (presence/absence, P/A) of benthic assemblages in no-take zones (NTZ) *vs* general use zones (GUZ) and among the three MPAs: Solitary Islands Marine Park (SIMP), Port Stephens-Great Lakes Marine Park (PSGLMP) and Batemans Bay Marine Park (BMP). Analyses were completed on all taxa, major groups, morpho-groups or morphology, including (+) or excluding (-) cover of substrata (see Table A in S1 File). Analyses used 9,999 permutations.

**Fig 4:**
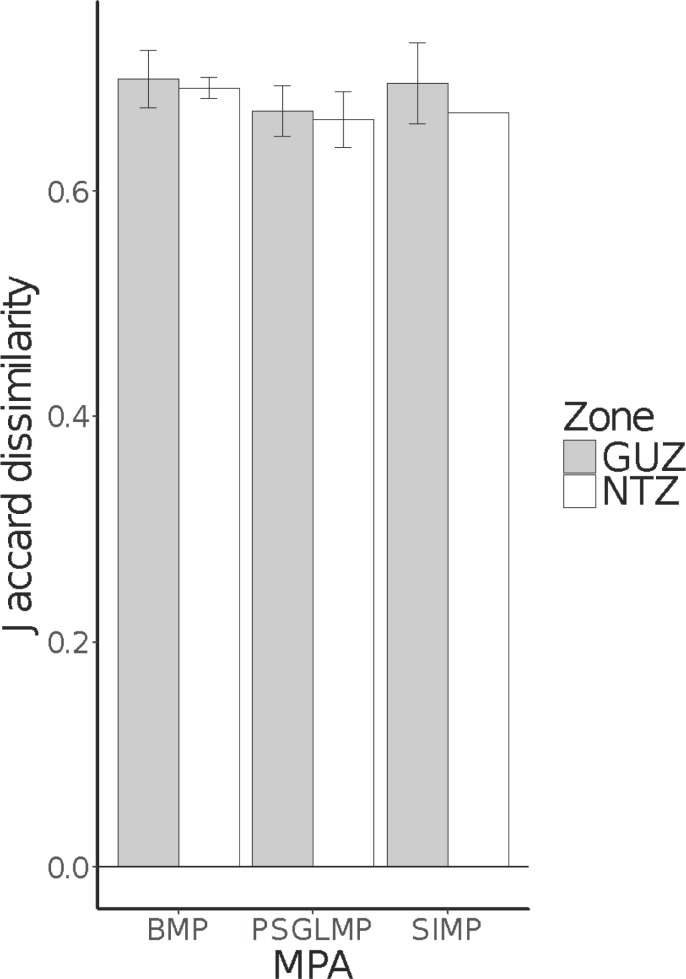
Mean Jaccard dissimilarities (+/- SE) across sites for each Zone and MPA combination.

The same pattern of no differences between zones but significant differences among MPAs was found in analyses based only on community composition (identity of taxa/groups only; Fig 3, Table 1), except for analyses at the level of major groups, where no differences between MPAs were observed (Table 1). Compositional variation, that is, variability in the identity of the taxa present in the community, quantified at the highest taxonomic resolution and pooled across all MPAs was significantly greater in GUZ than in NTZ, but no differences were found in analyses using broader taxonomic classifications (Table 2). This difference was not observed, however, when dissimilarities in the occurrence of taxa at the site level (rather than pooled across all MPAs) were compared and differences in the area sampled was accounted for, despite no effect of the latter (Fig 4, Table 3).

**Table 3:** Analysis of covariance of Jaccard dissimilarities among sites in each Zone and MPA combination, with area surveyed as a continuous covariate.

Source	df	SS	MS	F	p
Area	1	0.0000001	0.00000010	0.0001	0.9942
Zone	1	0.0006033	0.00060334	0.3434	0.5833
MPA	2	0.0041967	0.00209835	1.1942	0.3767
Zone x MPA	2	0.0001320	0.00006602	0.0376	0.9634
Residual	5	0.0087854	0.00175709		

### Prevalence and abundance of 16 dominant taxa

**Fig 5:**
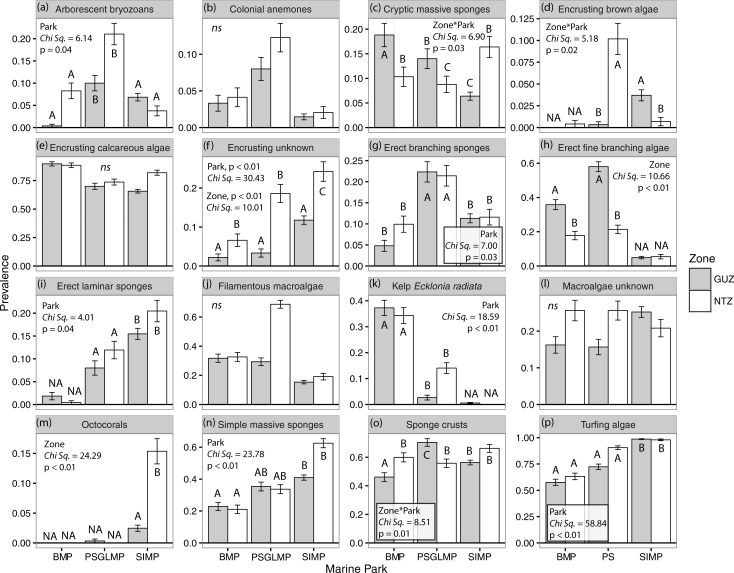
Mean (+/- SE) prevalence of the most prevalent taxa in each zoning type and MPA (SIMP NTZ n = 293, GUZ n = 892; PSGLMP NTZ n = 285, GUZ n = 300; BMP NTZ n = 242, GUZ n = 271). Prevalence is the probability of a taxa being present at a given zone and MPA, prevalence is independent across taxa so it does not need to add up to 1.0. Turfing algae = turf algae. NA: not included in analyses, ns = not significant. Capital letters (A, B, C) denote significant differences from post-hoc tests.

Analyses on the most prevalent (16) and abundant taxa (8), which together contributed 75–90% of the overall community dissimilarity between zoning types (Table B in S1 File), also showed an effect of zoning on several taxonomic groups of macroalgae, sponges and octocorals (Figs 4 and 5).

#### Prevalence between zones

At Solitary Islands, massive octocorals and cryptic massive sponges were more prevalent in NTZ than in GUZ (Table C in S1 File). There were non-significant trends of higher prevalence of both simple massive and erect laminar sponges in NTZ (Fig 5). At Port Stephens, erect fine branching macroalgae were more prevalent in GUZ. Contrastingly, brown macroalgae were more prevalent in NTZ. There was also a trend of higher prevalence of *E*. *radiata* and filamentous macroalgae in NTZ. Sponge crusts were also more prevalent at GUZ at Port Stephens; however, the opposite pattern was found at Batemans Bay (Table C in S1 File). At Batemans Bay, erect fine branching macroalgae were significantly more prevalent in GUZ than in NTZ. In both Batemans Bay and Port Stephens, arborescent bryozoans and unknown macroalgae appeared to be more prevalent in NTZ, but differences were not statistically significant (Fig 5).

#### Abundance between zones

**Fig 6:**
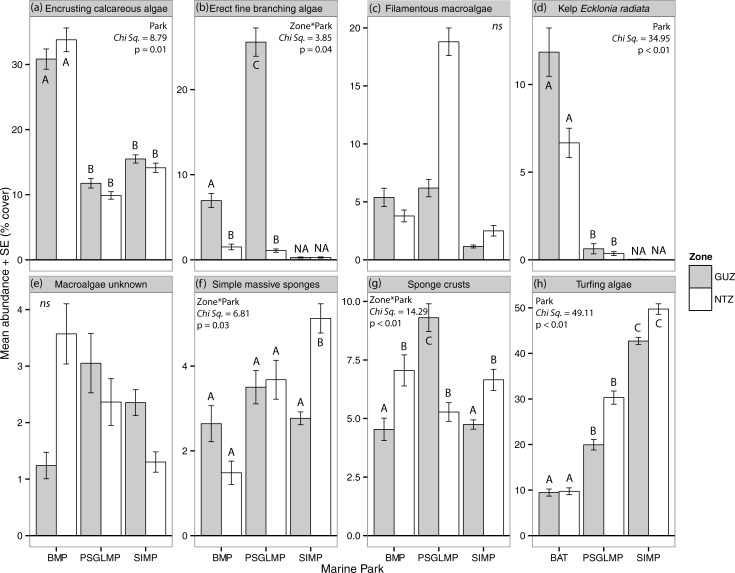
Mean (+/- SE) percentage cover of the most abundant taxa in each zoning type and MPAs (SIMP NTZ n = 293, GUZ n = 892; PSGLMP NTZ n = 285, GUZ n = 300; BMP NTZ n = 242, GUZ n = 271). NA: not included in analyses, ns = not significant. Capital letters (A, B, C) denote significant differences from post-hoc tests.

Massive sponges were significantly more abundant in NTZ than GUZ within Solitary Islands (Fig 5; Table D in S1 File). At Solitary Islands and Batemans Bay, sponge crusts were more abundant in NTZ, while at Port Stephens the opposite pattern was found. Erect fine branching macroalgae were more abundant in GUZ at Port Stephens and Batemans Bay (Table D in S1 File). In addition, there was a trend for greater coverage in NTZ of unknown macroalgae at Batemans Bay and of turfing algae in NTZ at Port Stephens and Solitary Islands (Fig 6).

#### Prevalence and abundance among MPAs

Differences among MPAs were driven by simple massive and erect laminar sponges, turfing algae and unknown encrusting biota, which were more prevalent and, in some cases, more abundant (algal turfs) at Solitary Islands than at Port Stephens or Batemans Bay (Fig 5; Table C in S1 File). Kelp and encrusting calcareous macroalgae were more prevalent and abundant at Batemans Bay than at Port Stephens or Solitary Islands (Figs 4 and 5 and Tables C and D in S1 File). Sponge crusts were more prevalent and abundant in GUZ at Port Stephens than at GUZ in other parks, while massive sponges were more abundant in NTZ at Solitary Islands than in NTZ in other parks (Fig 6).

## Discussion

This study generated an extensive dataset that will allow long-term assessments of epibenthic communities on rocky reefs on the inner continental shelf (25–50 m depth) of Southeastern Australia along a subtropical to temperate transition zone. Our first assessment of these benthic communities suggests that coarser groupings are suitable surrogates for more detailed taxonomic data in the monitoring of these MPAs, particularly when determining community-level effects. However, some ecologically important taxa, such as massive sponges, octocorals and brown macroalgae, were more prevalent and abundant in NTZ, despite the relatively recent zoning enforcement in these MPAs (5–10 years). This suggests the presence of potential positive short-term effects of MPAs on these communities. Given that observed differences were taxa-specific and may have not been evident using broader taxonomic classifications, more detailed classifications may be necessary to determine zoning effects on key taxa, such as dominant habitat-formers.

Analyses of taxa classified at the highest taxonomic level revealed a potential effect of NTZ on both the abundance and the prevalence of ecologically important benthic organisms, such as habitat-forming sponges and octocorals. Habitat-forming taxa were significantly more prevalent and abundant in NTZ at Solitary Islands, the MPA where NTZ have a longer implementation (10 years *vs* 5 years in Port Stephens and Batemans Bay). This might be explained by activities that result in physical disturbance in GUZ, such as trapping or anchoring, which may reduce habitat-forming invertebrates in these zones [50, 51]. Alternatively, the lower abundance of habitat-forming benthic biota in GUZ might also be influenced by an indirect effect of fishing and a release of mesopredators that consume benthic invertebrates as a consequence of reducing abundance of large piscivores [51,52, 53]. However, given that there are no data available from before the implementation of these MPAs and that sampling was done once, observed differences between NTZ and GUZ may be due to natural spatial variability rather than zoning effects. For instance, variability in the interconnectivity of source and sink reefs as well as the interactions between connectivity and habitat, which were not explicitly measured here, may affect the benthic community composition and structure [53, 54]. Repeated sampling at replicated NTZ and GUZ in each MPA will help detect zoning effects through time, if any [55].

If the differences found are influenced by zoning, this “short-term” effect on such ecologically key habitat-forming taxa is important because, while it may not be possible to directly protect these taxa from disturbances operating at the large scale, it may be possible to increase their prevalence, and thus resilience, by decreasing stressors operating at local scales through the establishment of NTZ [56, 57]. For instance, protecting top predators may reduce the abundance of consumers of benthic habitat-formers and can thus have a cascading, positive effect on these benthic organisms [58]. This may in turn contribute to an amelioration of the large-scale negative effects of climate change, which have been observed in recent years (*e*.*g*. massive bleaching event in SIMP during 2016) [59, 60]. For example, increases in the abundance and size of the generalist predatory fish, snapper (*Chrysophrys auratus*) is evident in the NTZ of the Solitary Islands [61]. This species is growth-overfished in NSW, with fishing mortality three times that of natural mortality [52]. Greater abundances of snapper in protected areas is likely to reduce the effect of herbivores that consume habitat-forming macroalgae [62].

There was no effect of management zone on benthic community structure or composition when considering multivariate changes in mean abundances. This pattern was consistent for all taxonomic classifications or groupings used, suggesting that broad classifications (e.g. Class in this case), which are faster and require less expertise, are accurate descriptors of community-level patterns. This has been shown to some extent by other studies in several marine systems, including intertidal mudflats, mangrove forests and subtidal soft-sediments [28]. Contrastingly, other studies argue that coarse taxonomic resolution of marine biota is not always adequate to detect changes in patterns at the community level [62]. The lack of differences between zones may be due to the fact that the NTZ are relatively young (5–10 years) and community-level effects can often take more than 20 years to be detected [54, 63, 64, 65]. Community structure differed between the MPAs, and this was expected given their latitudinal separation. Solitary Islands (30^o^ S) was dominated by massive sponges, corals and turf-forming algae, Port Stephens (32^o^ S) was dominated by branching sponges and fine branching algae (particularly in GUZ), and Batemans Bay (36^o^ S) was generally dominated by beds of *E*. *radiata*.

Human disturbances often affect not only average abundances of individual species (their means) or entire assemblages (multivariate centroids analogous to univariate means) through space and/or time, but also their variance (or multivariate dispersion for assemblages) [23]. Estimates of the community compositional variation across sites such as beta-diversity [66, 67] may be useful metrics to detect more rapid responses to the implementation of MPAs compared with mean changes in abundances [25]. Overall compositional variation, pooled across all MPAs, was greater in GUZ in analyses of taxa classified at the highest taxonomic accuracy possible, which may suggest some level of stress [68]. However, no differences were found in hierarchical analyses that considered the different spatial scales sampled within zones and MPAs and that took into account sampling intensity (i.e. differences in overall area sampled). This suggests there were no short-term effects on variability in community composition between zones and MPAs. Studies on effects of human disturbances on organisms typically focus on changes in their mean abundances, although spatial and/or temporal changes in their variances are neither less important nor less likely to be impacted [68, 69]. Future assessments of differences in both the means and variances of assemblages under different levels of protection/disturbance against the baseline described here may allow a more comprehensive, long-term assessment of the effectiveness of MPAs, particularly where multiple MPAs with differences in the time since establishment are compared [1, 10].

## Conclusion

Detecting the effects of zoning on benthic biodiversity and distribution requires statistical analyses on both the mean and variability in community composition and structure between management zones and MPAs. Hierarchical analyses that incorporate spatial and temporal variability and unbalanced sampling designs are required to assess the effectiveness of multiple MPAs over time. AUVs are effective tools to investigate effects of management on benthic communities across multiple spatial scales, and are especially useful to survey benthic communities in deep reefs where SCUBA surveys are constrained [18]. The data generated here can be used as the baseline against which to test zoning effects in the future.

Our results suggest that even young NTZ may have positive and potentially important effects on some dominant taxa on these rocky reefs, although repeated temporal sampling is needed to distinguish zoning effects from spatial variation. Nevertheless, observed differences were taxa-specific, and thus suggest that to detect effects of zoning, a finer taxonomic classification, such as the finest used here, may be necessary. It is expected that benefits of NTZ will increase as they are enforced through time [10]. A benthic community that supports habitat-formers is typically associated with an ecosystem that is capable of sustaining overall higher species diversity and abundance, which in turn can lead to greater resistance [7] and/or resilience [70,71]. More resistant and resilient ecosystems can have higher productivity and are often better prepared to withstand unmanageable disturbances, such as those related to climate change [8]. Thus, maintaining and promoting NTZ, as part of an adaptive and strategic marine spatial management plan, is crucial to conserve benthic communities and the services that they provide within and beyond MPAs boundaries.

## Supporting information

S1 File Supporting information. (DOCX)
