# Supplementary material for: Large-scale assessment of benthic communities across multiple marine protected areas using an autonomous underwater vehicle
Source: PLoS One. 2018 Mar 16;13(3):e0193711. doi: 10.1371/journal.pone.0193711 (PMC5856350; doi:10.1371/journal.pone.0193711)
Supplement: S1 File — (DOCX) [file pone.0193711.s001.docx]

Ferrari *et al.* – **Supporting Information**

**Table A.** List of taxa (with respective CATAMI classification codes), major taxonomic groups, morpho-taxa groups and morphology quantified during the surveys of three MPAs in New South Wales. Gray and white shading denote different high-taxonomic groups.

| **Major taxonomic group** | **Morpho-taxa** | **High taxonomic group** | **Morphology** |
| --- | --- | --- | --- |
| ascidian | Ascidian | Ascidians (35000000) (AS) | - |
| ascidian | Ascidian_stalk | Ascidians: Stalked (35000904) (ASS) | stalked |
| ascidian | Ascidian_stalk | Ascidians: Stalked: Colonial | stalked |
| ascidian | Ascidian_stalk | Ascidians: Stalked: Solitary (35000905) (ASSS) | stalked |
| ascidian | Ascidian_unstalk | Ascidians: Unstalked (35000901) (ASU) | encrusting |
| ascidian | Ascidian_unstalk | Ascidians: Unstalked: Colonial (35000903) (ASUC) | encrusting |
| ascidian | Ascidian_unstalk | Ascidians: Unstalked: Solitary (35000902) (ASUS) | encrusting |
| bacteria | Bacterial_mats | Bacterial mats (72000901) (BAM) | encrusting |
| bryozoan | Bryozoan | Bryozoa (20000000) (BRYO) | - |
| bryozoan | Bryozoan | Bryozoa: Hard (20000901) (BH) | encrusting |
| bryozoan | Bryozoan | Bryozoa: Hard: Encrusting (20000902) (BHE) | encrusting |
| bryozoan | Bryozoan | Bryozoa: Hard: Fenestrate (20000903) (BHF) | encrusting |
| bryozoan | Bryozoan | Bryozoa: Hard: Massive (20000904) (BHM) | encrusting |
| bryozoan | Bryozoan | Bryozoa: Soft (20000905) (BS) | filamentous |
| bryozoan | Bryozoan | Bryozoa: Soft: Dendroid (20000906) (BSD) | filamentous |
| bryozoan | Bryozoan | Bryozoa: Soft: Foliaceous (20000907) (BSF) | filamentous |
| coral | Coral | Cnidaria | - |
| anemone | Colonial_anemone | Cnidaria: Colonial anemones (11500901) (CA) | encrusting |
| anemone | Colonial_anemone | Cnidaria: Colonial anemones: Corallimorphs (11280000) (CACO) | encrusting |
| anemone | Colonial_anemone | Cnidaria: Colonial anemones: Zoanthids (11284000) (CAZ) | encrusting |
| hydrocoral | Hydrocoral | Cnidaria: Hydrocorals (11077000) (HYDCO) | branching |
| hydroid | Hydroid | Cnidaria: Hydroids (11001000) (HYDD) | filamentous |
| anemone | Anemone | Cnidaria: True anemones (11229000) (TRA) | branching |
| anemone | Anemone | Cnidaria: True anemones: Flytrap (11229901) (TRAFL) | encrusting |
| anemone | Tube_anemone | Cnidaria: Tube anemones (11164000) (TUA) | branching |
| coral | Octocoral | Corals: Black & Octocorals (11168901) (OCT) | - |
| coral | Octocoral_branching | Corals: Black & Octocorals: Branching (3D) (11168902) (OB) | branching |
| coral | Octocoral_branching | Corals: Black & Octocorals: Branching (3D): Fleshy (11168909) (OBF) | branching |
| coral | Octocoral_branching | Corals: Black & Octocorals: Branching (3D): Fleshy: Arborescent (11168911) (OBFA) | branching |
| coral | Octocoral_branching | Corals: Black & Octocorals: Branching (3D): Fleshy: Mushroom (11168910) (OBFM) | branching |
| coral | Octocoral_branching | Corals: Black & Octocorals: Branching (3D): Non-fleshy (11168903) (OBN) | branching |
| coral | Octocoral_branching | Corals: Black & Octocorals: Branching (3D): Non-fleshy: Arborescent (11168904) (OBNA) | branching |
| coral | Octocoral_branching | Corals: Black & Octocorals: Branching (3D): Non-fleshy: Bottle-brush (11168905) (OBNBB) | branching |
| coral | Octocoral_branching | Corals: Black & Octocorals: Branching (3D): Non-fleshy: Bottle-brush: Complex (11168907) (OBNBC) | branching |
| coral | Octocoral_branching | Corals: Black & Octocorals: Branching (3D): Non-fleshy: Bottle-brush: Simple (11168906) (OBNBS) | branching |
| coral | Octocoral_branching | Corals: Black & Octocorals: Branching (3D): Non-fleshy: Bushy (11168908) (OBNB) | branching |
| coral | Octocoral_encrusting | Corals: Black & Octocorals: Encrusting (11168919) (OE) | encrusting |
| coral | Octocoral_fan | Corals: Black & Octocorals: Fan (2D) (11168912) (OF) | fan |
| coral | Octocoral_fan | Corals: Black & Octocorals: Fan (2D): Fern-frond (11168913) (OFF) | fan |
| coral | Octocoral_fan | Corals: Black & Octocorals: Fan (2D): Fern-frond: Complex (11168915) (OFFC) | fan |
| coral | Octocoral_fan | Corals: Black & Octocorals: Fan (2D): Fern-frond: Simple (11168914) (OFFS) | fan |
| coral | Octocoral_fan | Corals: Black & Octocorals: Fan (2D): Rigid (11168916) (OFR) | fan |
| coral | Octocoral_massive | Corals: Black & Octocorals: Massive (11168920) (OM) | massive |
| coral | Octocoral_whip | Corals: Black & Octocorals: Whip (11168917) (OW) | branching |
| coral | stony_coral | Corals: Stony corals: Columnar | branching |
| coral | Stony_coral | Corals: Stony corals: Encrusting (11290908) (CEN) | encrusting |
| coral | Stony_coral | Corals: Stony corals: Foliose (11290907) (CFOL) | foliose |
| coral | Stony_coral | Corals: Stony corals: Massive (11290906) (CMAS) | massive |
| coral | Stony_coral | Corals: Stony corals: Massive (11290906): Mussidae (MMU) | massive |
| coral | Stony_coral | Corals: Stony corals: Solitary / mushroom (11290901) (CSO) | massive |
| coral | Stony_coral | Corals: Stony corals: Sub-massive (11290905) (CSM) | submassive |
| coral | Stony_coral | Corals: Stony corals: Tabulate (11290911) (CTAB) | tabulate |
| crustacea | Crustacea | Crustacea (27000000) (CRU) | encrusting |
| crustacea | Crustacea_barnacles | Crustacea: Barnacles (27500000) (CRB) | encrusting |
| echinoderms | Echinoderms | Echinoderms (25000000) (ECHI) | - |
| mobile-urchins | Sea-urchin | Echinoderms: Sea urchins: Regular urchins (25200901) (EURR) | - |
| mobile-urchins | Sea-urchin | Echinoderms: Sea urchins: Regular urchins: Centrostephanus: Rodgersii (25211001) (CROD) | - |
| macroalgae | Macroalga | Macroalgae (80300000) (MA) | - |
| macroalgae | Turf_macroalga | Macroalgae (80300000): Turfing algae (MATA) | turf |
| macroalgae | Calcareous_macroalga | Macroalgae: Articulated calcareous (80300911) (MAAC) | branching |
| macroalgae | Calcareous_macroalga | Macroalgae: Articulated calcareous: Red (80300913) (MAACR) | branching |
| macroalgae | Encrusting_macroalga | Macroalgae: Encrusting (80300926) (MAEN) | encrusting |
| macroalgae | Encrusting_macroalga | Macroalgae: Encrusting: Brown (80300927) (MAENB) | encrusting |
| macroalgae | Encrusting_macroalga | Macroalgae: Encrusting: Calcareous (80300929) (MAENC) | encrusting |
| macroalgae | Encrusting_macroalga | Macroalgae: Encrusting: Green (80300928) (MAENG) | encrusting |
| macroalgae | Encrusting_macroalga | Macroalgae: Encrusting: Red (80300929) (MAENR) | encrusting |
| macroalgae | Branching_macroalga | Macroalgae: Erect coarse branching (80300903) (MAEC) | branching |
| macroalgae | Branching_macroalga | Macroalgae: Erect fine branching (80300907) (MAEF) | branching |
| macroalgae | Filamentous_macroalga | Macroalgae: Filamentous / filiform (80300930) (MAF) | filamentous |
| macroalgae | Laminate_macroalga | Macroalgae: Laminate (80300918) (MALA) | encrusting |
| macroalgae | Large_canopy_macroalga | Macroalgae: Large canopy-forming: Brown (80300902) (MALCB) | canopy |
| macroalgae | Large_canopy_macroalga | Macroalgae: Large canopy-forming: Ecklonia: radiata (54080001) (ECKL) | canopy |
| macroalgae | Sheet_macroalga | Macroalgae: Sheet-like / membraneous (80300922) (MAM) | encrusting |
| mobile-chiton | Mullusc_chiton | Molluscs: Chitons (23100000) (MOCH) | - |
| sponges | Sponge | Sponges (10000000) (S) | - |
| sponges | Sponge_crust | Sponges: Crusts (10000901) (SCR) | encrusting |
| sponges | Sponge_erect | Sponges: Erect forms (10000912) (SE) | erect |
| sponges | Sponge_erect | Sponges: Erect forms: Branching (10000915) (SEB) | branching |
| sponges | Sponge_erect | Sponges: Erect forms: Laminar (10000913) (SEL) | branching |
| sponges | Sponge_erect | Sponges: Erect forms: Palmate (10000914) (SEP) | branching |
| sponges | Sponge_erect | Sponges: Erect forms: Simple (10000916) (SES) | branching |
| sponges | Sponge_hollow | Sponges: Hollow forms (10000909) (SH) | hollow |
| sponges | Sponge_hollow | Sponges: Hollow forms: Cups and alikes (10000910) (SHCA) | hollow |
| sponges | Sponge_hollow | Sponges: Hollow forms: Tubes and chimneys (10000911) (SHTC) | hollow |
| sponges | Sponge_massive | Sponges: Massive forms (10000903) (SM) | massive |
| sponges | Sponge_massive | Sponges: Massive forms (10000903): Barrels (SMB) | massive |
| sponges | Sponge_massive | Sponges: Massive forms (10000903): Cryptic (SMC) | massive |
| sponges | Sponge_massive | Sponges: Massive forms (10000903): Stalked (SMST) | massive |
| sponges | Sponge_massive | Sponges: Massive forms: Radially organised (10000905) (SMRA) | massive |
| sponges | Sponge_massive | Sponges: Massive forms: Simple (10000904) (SMSI) | massive |
| substrata | Substrate | Substrate (82001000) (SUB) | substrata |
| substrata | Substrate | Substrate: Consolidated (hard) (82001001) (SC) | substrata |
| substrata | Substrate | Substrate: Consolidated (hard): Boulders (82001003) (SCBOU) | substrata |
| substrata | Substrate | Substrate: Consolidated (hard): Cobbles (82001004) (SCCOB) | substrata |
| substrata | Substrate | Substrate: Consolidated (hard): Rock (82001002) (SCRO) | substrata |
| substrata | Substrate | Substrate: Unconsolidated (soft) (82001005) (SUN) | substrata |
| substrata | Substrate | Substrate: Unconsolidated (soft) (82001005), coarse-sand | substrata |
| substrata | Substrate | Substrate: Unconsolidated (soft) (82001005), Sand-mud (SUSA) | substrata |
| substrata | Substrate | Substrate: Unconsolidated (soft): Pebble / gravel (82001006) (SUPG) | substrata |
| worm | Worms | Worms: Echiura / Sipuncula: Echiura (17020000) (WESE) | - |
| worm | Worms | Worms: Penisworms (15400000) (WPE) | - |
| unknown1 | unknown1 | unknown1: Encrusting biota (ENBU) | encrusting |
| unknown2 | unknown2 | unknown2: Massive biota (MBU) | massive |
| unknown3 | unknown3 | unknown3: U | - |
| unknown4 | unknown4 | unknown4: Branching biota (BBU) | branching |

**Table B.** Contribution (%) of the 20 most abundant taxonomic groups to the dissimilarity between no-take and general use zones in each MPA (SIMPER; Bray-Curtis on square-root transformed covers). Univariate analyses were done on groups with either (a) mean prevalence >10% or (b) mean cover >5% in at least one Zone/MPA combination.

| **Group** | **SIMP** | **PSGLMP** | **BMP** |
| --- | --- | --- | --- |
| Arborescent bryozoans^(a)^ | 1.0 | 2.3 | 0.9 |
| Ascidians | 1.5 | - | - |
| Branching algae | 0.8 | - | - |
| Colonial anemones^(a)^ | - | 2.2 | - |
| Cryptic massive sponges^(a)^ | 2.3 | 2.6 | 4.0 |
| Encrusting algae | - | - | 0.9 |
| Encrusting brown algae^(a)^ | - | 2.1 | - |
| Encrusting calcareous algae^(a,b)^ | 15.4 | 9.8 | 18.3 |
| Encrusting unknown^(a)^ | 3.6 | 1.3 | - |
| Erect branching sponges^(a)^ | 2.1 | 3.0 | 1.4 |
| Erect fine branching algae^(a,b)^ | 1.2 | 16.3 | 7.9 |
| Erect laminar sponges^(a)^ | 3.4 | 1.1 | - |
| Filamentous macroalgae^(a,b)^ | 4.8 | 13.5 | 8.4 |
| Kelp *Ecklonia radiata*^(a,b)^ | - | 1.1 | 13.4 |
| Macroalgae unknown^(a,b)^ | 5.1 | 4.6 | 5.4 |
| Octocorals^(a)^ | 2.0 | - | - |
| Simple massive sponges^(a,b)^ | 8.8 | 6.1 | 4.9 |
| Sponge crusts^(a,b)^ | 10.0 | 8.9 | 9.9 |
| Stony corals | 2.4 | - | - |
| Turfing algae^(a,b)^ | 12.5 | 14.1 | 12.6 |
| **TOTAL** | **76.9** | **88.8** | **88.0** |

**Table C.** Likelihood-ratio tests for fixed terms in the binomial (Bernoulli) GLMM for prevalence of the dominant groups as a function of the fixed factors Zone (NTZ vs GUZ), MPA (SIMP vs PSGLMP vs BMP) and their interaction, with Plots nested in Sites as random effects.

|  |  | **Arborescent bryozoans** | | **Colonial anemones** | | **Octocorals^(a)^** | | **Encrusting unknown** | |
| --- | --- | --- | --- | --- | --- | --- | --- | --- | --- |
|  | *df* | *X*^2^ | *p* | *X*^2^ | *p* | *X*^2^ | *p* | *X*^2^ | *p* |
| Zone | 1 | 1.78 | 0.18 | 0.12 | 0.73 | 24.29 | **<0.01** | 10.01 | **<0.01** |
| MPA | 2 | 6.14 | **0.04** | 3.22 | 0.20 | - | - | 30.43 | **<0.01** |
| Zone:MPA | 2 | 1.87 | 0.39 | 0.01 | 0.99 | - | - | 0.39 | 0.82 |
|  |  | **Macroalgae unknown** | | **Turfing algae** | | **Encrusting brown macroalgae^(b)^** | | **Encrusting calcareous macroalgae** | |
|  | *df* | *X*^2^ | *p* | *X*^2^ | *p* | *X*^2^ | *p* | *X*^2^ | *p* |
| Zone | 1 | 1.28 | 0.26 | 1.38 | 0.24 | 0.15 | 0.70 | 0.42 | 0.52 |
| MPA | 2 | 1.69 | 0.43 | 58.84 | **<0.01** | 0.01 | 0.91 | 3.96 | 0.14 |
| Zone:MPA | 2 | 1.13 | 0.57 | 2.48 | 0.29 | 5.18 | **0.02** | 0.49 | 0.78 |
|  |  | **Erect fine branching macroalgae^(b)^** | | **Filamentous macroalgae** | | **Kelp *Ecklonia radiata*^(b)^** | | **Sponge crusts** | |
|  | *df* | *X*^2^ | *p* | *X*^2^ | *p* | *X*^2^ | *p* | *X*^2^ | *p* |
| Zone | 1 | 10.66 | **<0.01** | 2.15 | 0.14 | 1.02 | 0.31 | 0.93 | 0.34 |
| MPA | 2 | 1.04 | 0.31 | 1.41 | 0.50 | 18.59 | **<0.01** | 4.18 | 0.12 |
| Zone:MPA | 2 | 1.18 | 0.28 | 3.92 | 0.14 | 2.82 | 0.09 | 8.51 | **0.01** |
|  |  | **Erect branching sponges** | | **Erect laminar sponges^(b)^** | | **Cryptic massive sponges** | | **Simple massive sponges** | |
|  | *df* | *X*^2^ | *p* | *X*^2^ | *p* | *X*^2^ | *p* | *X*^2^ | *p* |
| Zone | 1 | 0.43 | 0.51 | 0.21 | 0.65 | 0.25 | 0.62 | 2.83 | 0.09 |
| MPA | 2 | 7.00 | **0.03** | 4.01 | **0.04** | 0.61 | 0.74 | 23.78 | **<0.01** |
| Zone:MPA | 2 | 0.71 | 0.70 | 0.34 | 0.56 | 6.90 | **0.03** | 5.41 | 0.07 |

^(a)^Only SIMP data were analysed because this group was found in <1% of the images in other parks.

^(b)^SIMP or BMP data were excluded from analyses on these groups as they were found in <2% of the images in one of these parks (*df*=1 for terms MPA and Zone:MPA).

**Table D.** Outcome of likelihood-ratio tests for fixed terms in the model for abundances of most the abundant groups. The number of observations of each group under 25 points in each image was modelled as a function of the fixed factors Zone (NTZ vs GUZ), MPA (SIMP vs PSGLMP vs BMP) and their interaction using a binomial GLMM, with Sites and Plots nested in Sites as random effects.

|  |  | Macroalgae unknown | | Turfing algae | | Encrusting calcareous macroalgae | | Erect fine branching macroalgae^(a)^ | |
| --- | --- | --- | --- | --- | --- | --- | --- | --- | --- |
|  | *df* | *X*^2^ | *p* | *X*^2^ | *p* | *X*^2^ | *p* | *X*^2^ | *p* |
| Zone | 1 | 1.25 | 0.26 | 1.44 | 0.23 | 0.10 | 0.76 | 26.90 | **<0.01** |
| MPA | 2 | 0.45 | 0.80 | 49.11 | **<0.01** | 8.79 | **0.01** | 1.16 | 0.28 |
| Zone:MPA | 2 | 0.56 | 0.76 | 0.11 | 0.95 | 0.35 | 0.84 | 3.85 | **0.04** |
|  |  | Filamentous macroalgae | | Kelp *Ecklonia radiata*^(a)^ | | Sponge crusts | | Simple massive sponges | |
|  | *df* | *X*^2^ | *p* | *X*^2^ | *p* | *X*^2^ | *p* | *X*^2^ | *p* |
| Zone | 1 | 1.07 | 0.30 | 0.01 | 0.98 | 0.95 | 0.33 | 0.28 | 0.60 |
| Park | 2 | 1.32 | 0.52 | 34.95 | **<0.01** | 5.31 | 0.07 | 8.10 | **0.02** |
| Zone:MPA | 2 | 3.18 | 0.20 | 0.75 | 0.39 | 14.29 | **<0.01** | 6.81 | **0.03** |

^(a)^SIMP data were excluded from analyses on these groups as they were found in very low abundance (<1% mean cover, many zeros) this park (*df*=1 for terms MPA and Zone:MPA).
